# Early Elevated Inflammatory Markers in SARS-CoV-2 Vaccinated Patients Are Associated with Reduced Mortality, Morbidity, and Lung Injury

**DOI:** 10.3390/life14111415

**Published:** 2024-11-02

**Authors:** Osama Khayat, Maamoun Basheer, Mayss Derawy, Nimer Assy

**Affiliations:** 1 Internal Medicine Department, Galilee Medical Center, Nahariya 2210001, Israel; mayss_derawy_97@hotmail.com (M.D.); nimera@gmc.gov.il (N.A.); 2 Azrieli Bar-Ilan Faculty of Medicine, Safad 1311502, Israel

**Keywords:** SARS-CoV-2, vaccines, CRP, IL-6, D-dimer, mechanical ventilation, immune system, inflammatory markers

## Abstract

**Background** The development of vaccines against SARS-CoV-2 has proved to be a highly successful strategy. In this work, the aim is to study the effects of the SARS-CoV-2 vaccine on the production of inflammatory markers and how this affect morbidity and mortality. Electronic medical record (EMR) data from 210 patients diagnosed with COVID-19 from November 2020 to June 2021 were collected. The admitted patients were divided into three groups, the one-dose vaccinated, two-dose vaccinated, and the non-vaccinated. All patients were moderate or severe in disease level as defined by the WHO classification. The results show that CRP was 101 ± 5.3, 97 ± 10.8, and 145 ± 17.3 (*p* < 0.05), fibrinogen 529 ± 16.3, 397 ± 33.8, and 610 ± 15 (*p* < 0.05), D-dimer 1244 ± 89, 1279 ± 297, and 1615 ± 224 (*p* < 0.05), ferritin was 1170 ± 122, 999 ± 202, and 1663 ± 409 (*p* < 0.05), IL-6 was 196 ± 12, 96 ± 5, and 580 ± 402 (NS), for the non-vaccinated, one-dose vaccinated, and two-dose vaccinated groups, respectively. The high level of CRP up to 150–200 mg/dL was more common among the surviving vaccinated patients. Oxygen supplementation, mechanical ventilation, and mortality were higher in the non-vaccinated group. Blood urea nitrogen (BUN) level was higher in the vaccinated patients, 25 ± 0.14 vs. 33 ± 6.15, respectively (*p* < 0.05). Inflammation markers were significantly higher in the vaccinated groups compared to non-vaccinated groups. On the other hand, extremely high levels of CRP (>200 mg/dL) were correlated with high mortality incidence.

## 1. Introduction

SARS-CoV-2 is a single-stranded RNA virus belonging to the family Coronaviridae. Humans infected with SARS-CoV-2 may develop COVID-19, which manifests across a wide spectrum of clinical severity ranging from a mild upper respiratory tract illness to a diffuse viral pneumonia-causing acute respiratory failure, with sequelae including acute lung injury, multi-organ dysfunction syndrome, and death [1,2,3].

The combination of the rapid development of effective vaccines against severe acute respiratory syndrome SARS-CoV-2 infection and their deployment in the general population has proved to be a highly successful strategy for reducing both viral transmission and disease burden [1,2,3].

The early initiation of a nationwide campaign resulted in the full vaccination (i.e., receipt of two vaccine doses) in more than half the population by the end of March 2021 [4,5]. Consequently, the incidence of COVID-19 was decreased [4,5]. Nevertheless, new concern variants (specifically, the delta variant and others) have led to a recent resurgence in confirmed infection and severe illness [6].

To address the challenge presented by the delta variant and to reduce the load on the health care system, the authorities approved the administration of a booster dose, first to high-risk populations, on 12 July 2021, and then to persons who were 60 years of age or older, on July 2021. Initial studies have suggested that a BNT162b2 booster dose increases the antibody neutralization level by a factor of approximately 10, on average, as compared with the level after a second dose [7].

High mortality was recorded in COVID-19 patients before the era of vaccines. The mortality is associated with a cytokine storm in the bodies of COVID-19 patients. The cytokine storm caused a lot of damage to the tissues in general and the lungs in particular [8,9]. An increase in inflammatory markers and cytokines was used as predictor of the severity of the disease in the second wave of the SARS-CoV-2 disease [8]. Furthermore, markers of biological waste such as BUN and urea were also an indicator of multi-system damage and even a parameter for mortality in those COVID-19 patients [8,9,10]. Therefore, high concentrations of these markers in patients before the era of vaccines showed a direct relationship to severe disease and mortality [8,9,10]. However, after the start of the SARS-CoV-2 vaccination campaign, the vaccinated patients showed a higher increase in inflammatory markers when exposed to SARS-CoV-2, in blood tests that were performed immediately upon their admission to the wards [8,9,10], in contrast to the non-vaccinated population, who showed a more moderate increase in inflammatory markers [8,9,10]. Therefore, the purpose of this work is to establish this hypothesis and check whether the excretion of inflammatory markers in the blood of vaccinated patients after exposure to SARS-CoV-2 was significantly higher than those found in the blood of non-vaccinated patients exposed to SARS-CoV-2. At the same time, we will try to study if these phenomena affected morbidity, mortality, and lung injury in the vaccinated and non-vaccinated patient groups.

## 2. Material and Methods

### 2.1. Study Population

This study is a “single center” and retrospective study. We collected and included data from the database of the Galilee Medical Center (GMC), a tertiary medical center. This study was approved by the Institutional Review Board (IRB) of the Galilee Medical Center, Nahariya, Israel.

Electronic medical record (EMR) data were collected from 210 patients diagnosed with COVID-19 from November 2020 to June 2021, in the Galilee Medical Center COVID-19 Department, Nahariya, Israel. The admitted patients were divided into three groups, the vaccinated and non-vaccinated groups as defined in the vaccination history. The vaccine used in the vaccinated group was the 0.3 mL/10 µg Pfizer-BioNTech vaccine, intramuscularly. All patients were moderate or severe as defined by the WHO COVID-19 severity classification. The previous status of the patient’s infection was not known. The EMR data that were collected for the groups included the symptoms and their duration before admission (fever, myalgia, dyspnea, and diarrhea), demographic background, past medical history and treatments, weight, BMI, blood laboratory tests (biochemistry, CBC, blood gases, blood type, coagulation tests), and inflammatory markers (CRP, IL-6, ferritin, D-dimer and fibrinogen). The association between mortality and inflammatory markers in the groups was studied by multivariate and univariate analysis.

### 2.2. Eligibility Criteria

#### 2.2.1. Inclusion Criteria

(1) Adult male or female ≥18 to ≤80 years of age;
(2) Admitted to the COVID-19 department due to proven SARS-CoV-2 infection per reverse transcription polymerase chain reaction (RT-PCR) assay of a pharyngeal sample (nasopharyngeal or oropharyngeal);
(3) Admitted to COVID-19 department from November 2020 to June 2021, in the Galilee Medical Center COVID-19 Department, Nahariya, Israel;
(4) Moderate and severe patients as defined by the WHO COVID-19 severity classification.

#### 2.2.2. Exclusion Criteria

(1) Pregnant (positive serum or urine test within 3 days before randomization) or nursing women;
(2) Male or female who was <18 or >80 years of age;
(3) Patients with septic shock;
(4) Patients whose vaccination status was undefined;
(5) Symptomatic patients with negative SARS-CoV-2 test.

### 2.3. Study Design

Retrospective analysis of the data was performed of the COVID-19 patients obtained from our electronic medical records for the COVID-19 patients. While the one-dose vaccination group was evaluated 2–3 weeks after the first dose of the vaccine, the two-dose vaccinated group was admitted to the COVID department at least 3 months after the second dose of the vaccine.

The parameters analyzed are symptoms and duration before admission (fever, myalgia, dyspnea, and diarrhea), demographic background, past medical history and treatments, weight, BMI, time to discharge or time to death, treatments upon hospitalization, blood laboratory tests (biochemistry, CBC, blood gases, blood type, coagulation tests, and inflammatory markers). Positive real-time PCR assays from nasal and nasopharyngeal samples did the confirmation of SARS-CoV-2 infection.

Blood samples were withdrawn from moderate and severe patients upon admission and incubated for 30 min at room temperature. After coagulation, the blood samples were centrifuged at 1500× *g* at 4 °C for 15 min and the serum was separated and aliquoted into 2-milliliter tubes and stored in a −80 °C freezer. For the cytokine testing, the serum was thawed on ice and the serum was pipetted into cryotubes until the assay was performed. To assess serum cytokine levels, the human high-sensitivity cytokine Luminex custom 8-plex kits (R&D Systems, Inc., Minneapolis, MN, USA) were used.

### 2.4. Ethics

Investigator Responsibilities Compliance with the Declaration of Helsinki and Good Clinical Practices. This study was performed under the Declaration of Helsinki (1964) as revised, most recently in Seoul (2008), US FDA regulations, and the ICH Guideline for Good Clinical Practice, E6 (R1). The ethics protocol number is 00096-23-NHR. Retrospective analysis of the data from our electronic medical record database was performed with the oversight of the ICH Guideline for Good Clinical Practice.

### 2.5. Statistical Methods

The WinSTAT program was used to resume the statistical analysis. The results were presented as mean ± SD. The frequency and corresponding diagnosis percentage were provided. Clinical and biochemical variables as independent variables were analyzed, and the univariate direct regression analysis and multivariate stepwise regression analysis were performed. Survival or death were the dependent variables. The significance level was less than 0.05. A *t* test was performed.

## 3. Results

### 3.1. Clinical Characteristics of the Vaccinated Versus Non-Vaccinated COVID-19 Patients

The data of 210 patients who suffered from COVID-19 were analyzed. All of these patients were admitted to the hospital. Of these patients, 145 were not vaccinated, 52 were two-dose vaccinated, and 13 of them were vaccinated with one dose of the vaccine.

There were no differences within the three groups in age, gender, BMI, comorbidities (diabetes, hypertension, lung disease, hemodialysis, and aspirin use), and the duration of symptoms before admission to the hospital (Table 1). No differences in the symptoms before admission (diarrhea, fever, and dyspnea) were found between the groups (Table 1). Nevertheless, the two-dose vaccinated group showed a decreased percentage of loss of taste versus the one-dose vaccinated patients and non-vaccinated patients, 9%, 46% and 28%, respectively (*p* < 0.05; Table 1).

There were no significant differences in the laboratory findings between the groups including hemoglobin, absolute neutrophil count, absolute lymphocyte count, and neutrophil to lymphocyte ratio, Platelet count, creatinine, triglycerides, high-density lipoproteins (HDL), and alanine aminotransferase (ALT) were tested.

Inflammatory markers were significantly higher in the two-dose vaccinated groups versus the one-dose vaccinated patients and non-vaccinated. The results showed that CRP was 101 ± 5.3, 97 ± 10.8, and 145 ± 17.3 (*p* < 0.05), fibrinogen 529 ± 16.3, 397 ± 33.8, and 610 ± 15 (*p* < 0.05), D-dimer 1244 ± 89, 1279 ± 297, and 1615 ± 224 (*p* < 0.05), ferritin was 1170 ± 122, 999 ± 202, and 1663 ± 409 (*p* < 0.05), IL-6 was 196 ± 12, 96 ± 5, and 580 ± 402 (NS), for the non-vaccinated, one-dose vaccinated, and two-dose vaccinated groups, respectively (Table 1). Oxygen supplement on admission was higher but not significant in the non-vaccinated group versus the vaccinated group, 70% vs. 60%, respectively. Mechanical ventilation was also higher in the non-vaccinated group, with 68 (46%) of non-vaccinated patients needing mechanical ventilation versus 20 (38%) patients in the two-dose vaccinated group. The mortality was higher in the non-vaccinated group than in the vaccinated group; 37 patients died in the non-vaccinated group versus 21 in the vaccinated group (Table 1). Another significant difference between the non-vaccinated and vaccinated groups was in BUN level, 25 ± 0.14, 21 ± 2.5, and 33 ± 6.15 (*p* < 0.05), for the non-vaccinated, one-dose vaccinated, and two-dose vaccinated groups, respectively (Table 1).

### 3.2. The Effect of the SARS-CoV-2 Vaccine on the Frequency of Lung Injury, Mechanical Ventilation, and Mortality

**Figure 1:**
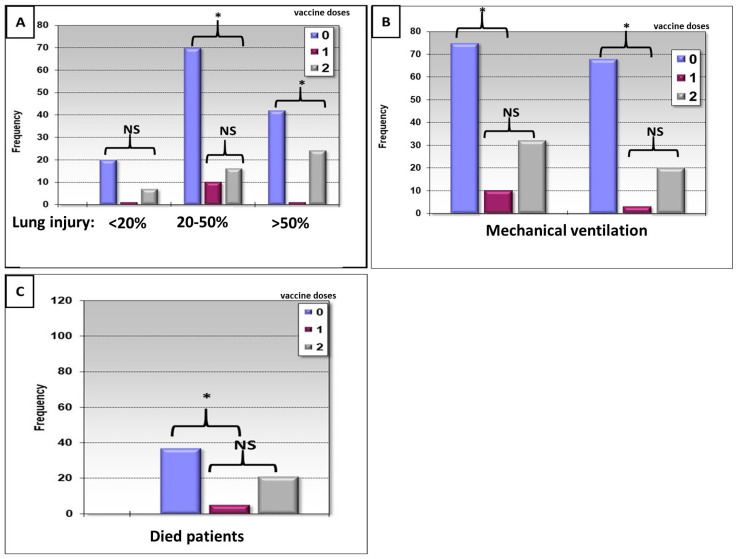
The early effect of the SARS-CoV-2 vaccine (non-vaccinated, one-dose vaccinated, and two-dose vaccinated) on the frequency of lung injury (**A**), frequency of mechanical ventilation usage (**B**), and frequency of mortality (**C**). The frequency of lung injury (**A**), mechanical ventilation (**B**), and mortality (**C**) is shown. 0 is non-vaccinated patients, 1 is one-dose SARS-CoV-2 vaccinated patients, and 2 is two-dose vaccinated patients. * *p* < 0.05. The statistical comparison was performed between non-vaccinated patients (0) and one-dose vaccinated patients and on the other hand between two doses of vaccination and 0 and 1 dose. Since the statistical behavior of one dose of the vaccine or no dose was similar, the statistical marking in the graph shows the two vaccine dose group versus the other two groups. NS: not significant.

The effect of the SARS-CoV-2 vaccine on lung injury (Figure 1A), frequency of mechanical ventilation usage (Figure 1B), and frequency of mortality (Figure 1C) was studied in COVID-19 patients.

The frequency of severe lung injury was significantly shown in non-vaccinated patients, while vaccinated patients showed a lower rate of lung involvement (Figure 1A). The SARS-CoV-2-mediated lung injury was divided into three classes: the first one was involvement up to 20% lung injury, the second class was 20–50% of lung involvement, and the third class was more than 50% lung involvement. In the three subclasses of lung injury, no vaccinated patient suffered from more lung injury. Mechanical ventilation and mortality were more frequently in non-vaccinated (Figure 1B,C) than in one-dose vaccinated patients.

### 3.3. The Survival Frequency of Patients Who Survived and Those Who Died According to the Levels of the Inflammatory Marker CRP and Fibrinogen

**Figure 2:**
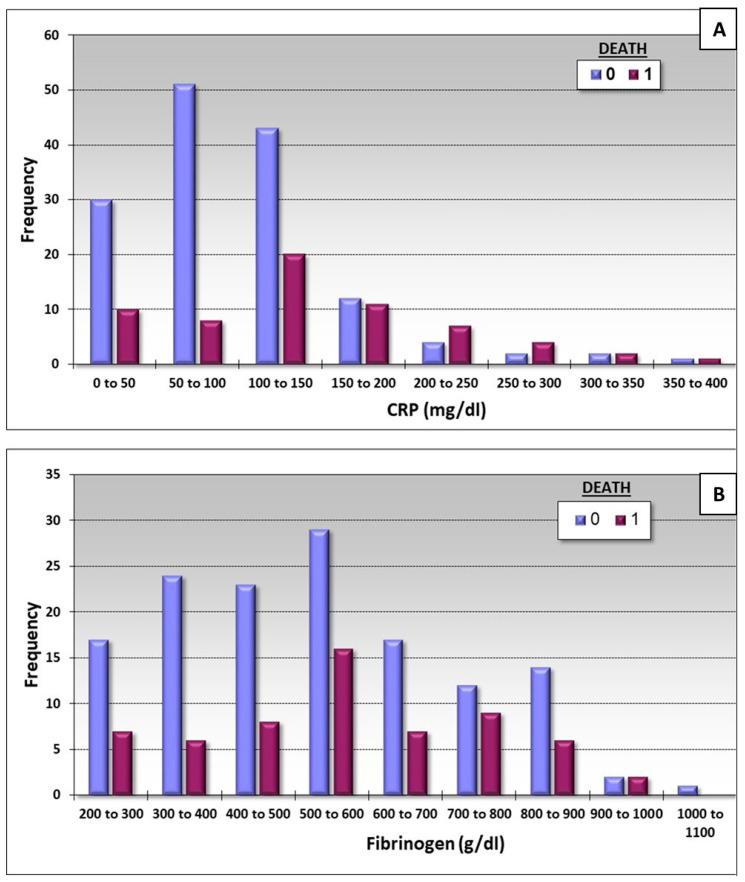
The early survival frequency of the patients who survived and died according to the levels of CRP (**A**) and fibrinogen (**B**). The blue columns are the survival patients (0) and the violet ones are the non-survivals (1).

The inflammatory marker CRP was higher specifically in patients who were two-dose vaccinated. On the other hand, survived patients show high level of CRP upon admission up to a certain level of about 150–200 mg per dL, while above this level, patients who died show higher levels of CRP than those who survived (Figure 2A).

There were more survived patients versus none survived patients in all fibrinogen concentration levels (Figure 1). This demonstrates that patients who survived had higher levels of fibrinogen relative to those who died (Figure 2B).

## 4. Discussion

The clinical presentation of acute respiratory syndrome infection has a wide range of severity, from a mild upper respiratory tract inflammation to a diffuse viral pneumonia [1,2,3]. According to the NIH management guidelines, 80% of COVID-19 patients worldwide were classified as mild (fever, cough, malaise), 14% as severe (pneumonia and hypoxemia) and 5% as critical illness -such as septic shock and acute respiratory distress syndrome (ARDS) [1,2,3]. The development of effective vaccines against severe SARS-CoV-2 infection has proved to be a highly successful strategy for reducing both viral transmission and disease burden. The purpose of this work is to check whether the excretion of inflammatory markers in the blood of vaccinated patients after exposure to the SARS-CoV-2 is higher than those found in the blood of non-vaccinated patients exposed to the SARS-CoV-2 and how these early elevations in these inflammatory markers correlate with morbidity, mortality and lung injury.

The concentration of the different markers of inflammation (IL-6, CRP, ferritin, D-dimer, and fibrinogen) in COVID-19 was significantly higher in the two-dose vaccinated patients versus one dose and non-vaccinated patients. Mechanical ventilation and mortality were higher in the non-vaccinated group than in the vaccinated ones. There are no differences in the symptoms before admission (diarrhea, fever, and dyspnea) between the different vaccinated and non-vaccinated groups. Nevertheless, the two-dose vaccinated group showed a decrease in the percentage who suffered from a loss of taste. BUN also increased significantly in the vaccinated groups.

The vaccine was the main turning point in the SARS-CoV-2 epidemic. Many research studies show that vaccines are effective in preventing symptomatic COVID-19 [11,12,13]. The effectiveness is high for the more serious outcomes, such as hospitalization, severe illness, and death [13,14]. Our results confirmed these findings. Vaccines reduce mortality and the severity of the disease (Table 1). However, we also show that vaccinated patients produce a high concentration of inflammatory markers, more than non-vaccinated and one-dose vaccinated patients.

The systemic inflammatory response to the SARS-CoV-2 infection is a hallmark of COVID-19. Most hospitalized patients with COVID-19 have abnormal inflammatory biomarkers [15]. Before the COVID-19 global pandemic, up to 90% of all marked elevations in CRP concentration attributed to an infectious etiology, most often from bacterial pathogens. However, after the pandemic, increased CRP concentration shown to be significantly elevated in COVID patients [15,16,17,18,19,20,21,22]. An association between higher CRP concentrations and greater disease severity in COVID-19 recorded previously [15,16,17,18,19,20,21,22]. Recent reports also identified associations between CRP concentrations and respiratory failure requiring mechanical ventilation, with a nearly five-fold greater risk of acute respiratory distress syndrome [23,24].

Other studies also showed that D-dimer was independently associated with adverse events [24].

Our study showed that vaccinated patients who were infected with the SARS-CoV-2 virus presented at admission with highly significant levels of inflammatory markers (IL-6, ferritin, fibrinogen, D-dimer, and CRP). Nevertheless, non-vaccinated patients showed low levels of these inflammatory markers. The explanation is that immunogenic properties of the SARS-CoV-2 vaccine. There is concern that the vaccine would activate the immune system immediately after exposure to the new pathogen (SARS-CoV-2) [25,26,27,28]. The hyper-activation of the immune system is the main preparation of the immune system following SARS-CoV-2 infection. The cytokine secretion and inflammatory markers released are some of the immune system’s fighting strategies to eliminate the virus out of the body. The maximal preparation is unique for the people who vaccinated. On the other hand, these parameters positively correlated with survival and mortality (Figure 1 and Figure 2). Traditionally, markers such as interleukin-6 (IL-6), C-reactive protein (CRP), and tumor necrosis factor-alpha (TNF-α) have been associated with poor outcomes in unvaccinated COVID-19 patients. However, in vaccinated individuals, these elevated inflammatory responses may represent an enhanced immune activation rather than pathological inflammation. A study on SARS-CoV-2 breakthrough infections found that vaccinated patients displayed significantly higher levels of inflammatory markers such as IL-6 and CRP compared to unvaccinated individuals [29]. Importantly, these markers were associated with faster viral clearance and better clinical outcomes, including reduced morbidity and mortality [29]. This suggests that the immune systems in vaccinated individuals are more adept at controlling viral replication, possibly due to a primed and more efficient inflammatory response. The vaccination appears to act as a modulator of the immune system, enhancing the response without tipping into harmful hyperinflammation. Furthermore, a study investigating the correlation between inflammatory cytokines and antibody levels in vaccinated patients found that higher levels of IL-6 and CRP linked to increased SARS-CoV-2-specific antibody production. This correlation between cytokine and antibody levels suggests that the inflammatory response plays a critical role in amplifying the body’s defense against the virus, contributing to better recovery and fewer complications [30].

Moreover, findings from these studies align with the broader understanding of vaccine-induced immunity. The elevation in inflammatory markers post-vaccination may indicate a more controlled, adaptive immune response, helping to clear the infection more effectively while reducing lung injury and other severe outcomes. This hypothesis is further supported by the research on T-cell responses in vaccinated individuals, where the balance of pro-inflammatory cytokines like TNF-α and IFN-γ was shown to contribute to better lung function outcomes, even in the presence of persistent symptoms such as those seen in post-acute sequelae of SARS-CoV-2 [31,32].

Obese women have higher levels of inflammatory markers compared to men due to several theoretical explanations [33,34,35,36]. The study group (two dose-vaccinated patients) were mostly males and with normal BMI relative to those who were not vaccinated or vaccinated with a single dose (Table 1). This strengthens our results which emphasize that even though the unvaccinated patients have a high BMI and female gender and we would expect them to have high levels of inflammatory markers, the “thin” vaccinated patients still have more inflammatory markers, which strengthens our theory that claims that vaccinated patients produce more inflammatory markers as an initial stage of protection against the virus.

Our results show that up to a certain level of CRP (150–200 mg) were frequent in survival patients, but higher levels correlated with mortality (Figure 2). This could explain how a non-vaccinated patient who mostly suffers from being overweight produces cytokine storms, which introduced by a high level of cytokines; this phenomenon mediates a high risk of mortality. Figure 2 demonstrates that this is true up to a certain level of CRP (150–200); beyond that level, patients did not survive, and this could be explained by the fact that such high levels of CRP are probably part of a cytokine storm, a very dangerous situation that usually causes the death of the patients. A cytokine storm is a life-threatening systemic inflammatory syndrome involving elevated levels of circulating cytokines and immune-cell hyperactivation [8,36]. The cytokines associated with COVID-19 are interleukin-1β, interleukin-6, IP-10, TNF, interferon-γ, macrophage inflammatory protein (MIP) 1α and 1β, VEGF, and very high CRP [8,36]. There is no clear cutoff for the concentration of the cytokine to determine the existence of a cytokine storm [8,36]. It should be emphasized that cytokines may be both a key component of the cytokine storm and an essential factor in the antimicrobial response [8,36]. Our study shows that survival was related to a high concentration of fibrinogen, as seen in Figure 2. The concentration of fibrinogen was higher in the group of patients who survived and those who were two-dose vaccinated. Several important differences in therapeutic considerations should be noted between the COVID-19-associated cytokine storm in vaccinated and non-vaccinated groups.

Overall, these findings suggest that the elevation of inflammatory markers in vaccinated patients represents a controlled immune response that reduces the risk of severe outcomes, in contrast to the uncontrolled hyperinflammation often seen in severe cases of COVID-19 in non-vaccinated individuals. Future research should aim to elucidate the mechanisms by which vaccination primes the immune system.

Our study also shows that the BUN levels increased significantly in the vaccinated group. Likewise, markers of biological waste such as BUN and urea are considered as an indicator of multi-system damage and even as a parameter for mortality in those patients [8,9]. Therefore, high concentrations of these markers in patients before the era of vaccines showed a direct relationship with severe disease and mortality [8,9,10]. The increased blood urea nitrogen (BUN) levels observed in vaccinated COVID-19 patients may be also linked to several physiological responses triggered by the virus and vaccination. One key factor is the impact of COVID-19 on the kidneys, which has been well documented in both vaccinated and non-vaccinated patients. COVID-19 can cause kidney damage directly by infecting kidney cells or indirectly through hypoxia (low oxygen levels), inflammation, and the formation of blood clots [37]. Vaccination itself, while protective, can still induce immune responses that might exacerbate underlying conditions like kidney dysfunction, especially in patients with pre-existing hypertension, diabetes, or chronic kidney disease. Moreover, vaccines trigger robust immune reactions, including the production of cytokines, which may stress the kidneys, particularly in patients already vulnerable due to COVID-19’s effect on the kidneys. The increased inflammation could contribute to elevated BUN levels as the kidneys struggle to filter waste during such systemic responses [38]. This area warrants further research. Understanding the interplay between immune responses, kidney function, and vaccine impact is crucial in managing patients effectively.

The most common general symptoms frequently encountered with COVID-19 include dry cough, fever, muscle pain, anorexia, dyspnea, fatigue, and loss of taste and smell sensations [39]. These symptoms may progress to cytokine storm syndrome, insomnia, and even respiratory failure [1,39]. Interestingly, the SARS-CoV-2 vaccine reduced significantly the virus-related symptoms. Our study showed that the vaccine decreased significantly the percentage of patients who suffered from loss of taste sensation and other symptoms (Table 1).

In the future, we intend to continue studying the direct effect of these parameters on long-term SARS-CoV-2 complications such as morbidity, mortality, and long COVID. On the other hand, we want to continue studying the mechanism underlying the preventative effect of these parameters.

## 5. Limitation

This research was performed at the beginning of the SARS-CoV-2-vaccine era. Its purpose was to study the direct effect of vaccines on the production of inflammatory markers and their direct effect on morbidity and mortality. It is important to emphasize this again. A second disadvantage was the size of the sample; it was a relatively small sample that explains important points and therefore a larger sample is required to substantiate these points and results. The mechanism underlying the effect of inflammatory markers on mortality and morbidity was not discussed in this study.

## 6. Conclusions

Inflammatory markers were significantly higher in the vaccinated groups compared to non-vaccinated groups. The secretion of cytokines and the release of inflammatory markers are part of the fighting strategies of the immune system. Extremely high levels of inflammatory markers are compatible with a cytokine storm, a very dangerous state that causes a high mortality rate.

## Figures and Tables

**Table 1:** Clinical characteristics of the vaccinated (one dose and two doses) versus non-vaccinated patients with SARS-CoV-2 infection. The early effect of the vaccine on mortality and severity is presented. The concentration of the different inflammatory markers (IL-6, CRP, ferritin, D-dimer, and fibrinogen) in COVID-19 patients was measured. The statistical comparison was performed between non-vaccinated patients (0) and one dose of vaccinated patients and on the other hand, between two doses of vaccination and 0 and 1 dose. The statistical comparison was performed between nonvaccinated patients (0) and one dose of vaccinate patients and on the other hand between two doses of vaccination and 0 and 1 dose. Since the statistical behavior of one dose of the vaccine or no dose was similar, the statistical marking in the table shows the two vaccine doses group versus the other two groups. NS: not significant (*p*-value > 0.05).

Variable	Non-Vaccinated	Vaccinated (One Dose)	Vaccinated (Two Dose)	*p*-Value
Total	***n* = 145**	***n* = 13**	***n* = 52**	
Age	62 ± 1.8	66 ± 6.3	66 ± 5.5	NS
Male (%)	47%	39	58	NS
BMI	30 ± 0.4	27 ± 1.6	25 ± 0.96	NS
**Comorbidities %**				NS
Diabetes (%)	43	46	53	NS
Hypertension (%)	44	69	61	NS
Lung disease (%)	38	30	25	NS
Hemodialysis (%)	21	23	13	NS
Aspirin use (%)	22	30	23	NS
**Symptom’s duration (days)**	4 ± 0.25	3.9 ± 0.9	4 ± 0.57	NS
Loss of taste (% of total)	28	46	9	0.006
Fever (% of total)	55	61	50	NS
Diarrhea (% of total)	16	15	15	NS
Dyspnea (% of total)	70	76	73	NS
**Lab Findings upon admission**				NS
Hemoglobin (mg/dL)	12.0 ± 0.2	12 ± 0.5	11 ± 0.5	NS
Absolute neutrophil count (×10^3^/µL)	9 ± 0.1	7 ± 0.33	9 ± 1.15	NS
Absolute lymphocyte count (×10^3^/µL)	1.6 ± 0.025	1.7 ± 0.2	1.4 ± 0.4	NS
Neutrophil to lymphocyte ratio (NLR)	10 ± 0.67	2.6 ± 0.5	7.4 ± 1	NS
Platelet (×10^3^/µL)	223± 0.87	181 ± 26.6	197 ± 15	NS
BUN (mg/dL)	25 ± 0.14	21 ± 2.5	33 ± 6.15	0.045
Creatinine (mg/dL)	1.4 ± 0.08	1.34 ± 0.16	2.3 ± 0.7	NS
Triglycerides (mg/dL)	174 ± 6.6	173 ± 10.2	150 ± 25.9	NS
HDL (mg/dL)	28 ± 1.25	25 ± 1.38	25 ± 1.9	NS
C-reactive protein (CRP) (mg/dL)	101 ± 5.3	97 ± 10.8	145 ± 17.3	0.005
Ferritin	1170 ± 122	999 ± 202	1663 ± 409	0.03
D-dimer	1244 ± 89	1279 ± 297	1615 ± 224	0.04
Fibrinogen	529 ± 16.3	397 ± 33.8	610 ± 15	0.0049
IL-6	196 ± 12	96 ± 5	580 ± 440	NS
ALT	48 ± 3.9	67 ± 9.4	51 ± 20	NS
O_2_ supplement (% of total)	70	69	60	NS
Mechanical ventilation (% of total)	68 (46%)	3 (23%)	20 (38%)	NS
% of lung injury	47 ± 1.75	37 ± 3.8	47 ± 4	NS
Death (N)	37	5	21	0.0499

## Data Availability

The data are found at the internal medicine department at Galilee Medical Center, Nahariya, Israel, and will be available upon reasonable request.
